# Dental management of patients with sensory impairments

**DOI:** 10.1038/s41415-022-5085-x

**Published:** 2022-10-28

**Authors:** Tasnim Aniqa Ahmed, Natalie Bradley, Stephanie Fenesan

**Affiliations:** 4141510451001 Foundation Dentist, Oak Hill Dental and Implant Centre, 101 Oak Hill, Woodford Green, IG8 9PF, UK; 4141510451002 Specialist in Special Care Dentistry, Whittington Health Trust, Magdala Ave, London, N19 5NF, UK; 4141510451003 Specialty Registrar, Sedation and Special Care Dentistry, Guy´s and St Thomas´ NHS Foundation Trust, Floor 26, Tower Wing, London, SE1 9RT, UK

## Abstract

This article discusses the different types of sensory impairments and their aetiology. It considers how the oral health status in patients with sensory impairments is impacted by their disability and the barriers these patients face in dental care. It also discusses legislation relevant to dental care professionals when caring for patients with disabilities, including the Mental Capacity Act (2005), the Equality Act (2010) and the Accessible Information Standard (2016). Finally, it provides recommendations to dental care professionals on how they can best manage patients with sensory impairments and communicate with them effectively in order to provide them with quality dental care.

## Key points

- Provides an overview of the prevalence and causes of sensory impairments.
- Describes the legislative requirements for dental practitioners when managing these patients and the oral health of people with sensory impairments.
- Suggests reasonable adjustments that dental practitioners can make when caring for this patient group.

## Introduction

According to the Equality Act 2010, a person has a disability if they have a 'physical or mental impairment and the impairment has a substantial and long-term adverse effect on their ability to carry out normal day-to-day activities'.^1^ The way we communicate with our patients often greatly relies on our senses, such as our vision and hearing; however, for some patients, they may lack the use of one, or the other, or both. In this article, we will focus on patients with sensory impairments and how to manage those who have them.

In the UK, there are approximately two million people who have sight loss, with 360,000 of these being registered as blind or partially sighted.^2^ Additionally, there are 11 million people who are deaf or hard of hearing and 150,000 who use British Sign Language (BSL).^3^ Hearing impairments can be congenital, inherited or acquired throughout life. Furthermore, there are approximately 400,000 patients who suffer from deafblindness (deafness and blindness), which can be referred to as a dual sensory impairment.^4^ Types of sensory impairments and their causes can be seen in Table 1.

**Table 1:** Types of sensory impairment, types and cause^19^^,^^40^^,^^43^^,^^44^^,^^45^

Impairment	Hearing	Visual	Dual sensory or deafblindness
Definition	An impairment which occurs when there is a problem with, or damage to, one or more parts of the ear	A visual disability which cannot be corrected by spectacles or lenses	Impairments affecting both vision and hearing where the deterioration or progressive loss of their sight and/or hearing causes a significant functional impact on one or more of the following: Communication Access to information Mobility
Types	Conductive hearing loss - occurs due to a problem with the outer or middle ear Sensorineural hearing loss - occurs due to damage to the inner ear or the auditory nerve Mixed hearing loss - occurs when a patient has both conductive and sensorineural hearing problems Central hearing loss - occurs when the cochlea is working properly but other areas of the brain are not Auditory processing disorders - patients have difficulty hearing in a noisy environment	Partial sightedness - people who cannot clearly see how many fingers are being held up at a distance of six metres or less, even with glasses or lenses Blindness - people who are unable to clearly see how many fingers are being held up at a distance of three metres or less, even with glasses or lenses	People who are hearing and sight impaired from birth or early childhood Those blind from birth or early childhood who subsequently acquire a hearing loss that has a significant functional impact Those who are deaf from birth or early childhood who subsequently acquire a significant visual loss Those who acquire a hearing and sight impairment later in life that has a significant functional impact
Causes	Congenital deafness Head injury Exposure to loud noise Age-associated deafness Tinnitus Tumours Infections, for example, mumps, measles, rubella, repeated otitis media Drugs, for example, some antibiotics and chemotherapy drugs	Cataract Undercorrected refractive error Glaucoma Age-related macular degeneration Diabetic retinopathy Corneal opacity Infections - rubella, toxoplasmosis, trachoma Tumours Hemianopia Nystagmus High degree myopia Uveitis Syndromes, for example, Ehlers Danlos, Marfan, Treacher-Collins, Sjögren's, Behcet's Drugs for example, quinine, methanol, phenothiazines Trauma	Congenital, for example, due to contraction of rubella during the first trimester of pregnancy, premature birth, difficulties in labour Acquired, for example, Usher syndrome, accident and illness, aging

The Accessible Information Standard (AIS) is a resource which was introduced by NHS England in 2016.^5^ It aims to ensure that those with a disability are provided with information in a way that they can easily understand, allowing them to better communicate with the healthcare professionals they encounter. All organisations offering NHS care are legally required to follow the Standard (commenced 1 August 2016), including dental services. The Standard sets out a consistent approach to identifying, recording, flagging, sharing and meeting the information and communication support needs of patients, service users, carers and parents with a disability, impairment or sensory loss. Key points in AIS for dental providers to be aware of are listed in Box 1.

When we communicate, there are two types of skills that we use - expressive and receptive. Expressive communication is that in which a message is sent, either to begin communication or to respond to someone else's message. Receptive communication is the receipt of a message given to you by someone else. Each individual has different expressive and receptive skills and this includes patients with sensory impairments. For example, a person with a hearing impairment may rely on sign language as part of their receptive communication skills and use sign language and/or speech as part of their expressive communication skills.^6^

Some patients - for example, those with learning disabilities or older people living in care homes - may have documentation that can support professionals in understanding the information and communication support needs of said patient, such as health or communication passports. These can also be invaluable sources of information to assist dental teams in communicating effectively with patients with sensory impairments. These can include information on the patients' preferred method of communication, or adjustments that can be made to communication to aid understanding. Examples include the use of loop systems, braille, or Makaton or BSL, among others.

**Box 1** AIS requirements for healthcare providers*

- Professionals must identify and record the information and/or communication needs of their patients and service users where such needs relate to or are caused by a disability, impairment or sensory loss
- Staff should proactively prompt individuals to identify that they have information and/or communication needs and support them to describe the type of alternative format and/or support that they need
- Electronic and paper-based recording and administration systems must enable recording of information and communication needs
- Organisations must ensure that data recorded about individuals' information and communication support needs is current
- Organisations must ensure that information about individuals' information and/or communication support needs is shared as a routine part of any referral, discharge and handover
- Organisations must ensure that patients, service users, carers and parents with information needs are sent or otherwise provided with information, including correspondence, in formats which are appropriate, accessible and that they are able to understand and have accessible contact methods
- Organisations must ensure that patients, service users, carers and parents are provided with appropriate communication support, including using aids or equipment and/or by staff making adjustments to their behaviour to enable effective communication
- Organisations must ensure that communication professionals (including BSL interpreters and deafblind manual interpreters) used in health and social care settings have:
  - Appropriate qualifications
  - Disclosure and Barring Service (DBS) clearance
  - Signed up to a relevant professional code of conduct.
- Organisations should ensure that patients, service users, carers and parents with information and/or communication support needs are given a longer appointment where this is needed to support effective communication/the accessible provision of information
- Individuals must be encouraged and enabled to provide feedback about their experience of receiving information in an appropriate format or communication support, including having access to an accessible complaints policy.

*Information taken from^42^

## Aims

- To describe the different types of sensory impairments and their aetiology
- To raise awareness of resources available to dental care professionals, which they can refer to for guidance when treating patients with sensory impairments
- To explore the barriers to dental care for these patients and how to manage and communicate with them effectively.

## The oral health status of people with sensory impairments

The oral health of this group of patients is generally poorer than the general population. Many sensory impairments are associated with other medical conditions or disabilities. Poor oral health for these groups will be multifactorial, as a result of:

- The direct impact of their sensory impairment, for example, difficulties visualising when undertaking oral hygiene^7^
- The impact of any associated medical conditions, such as diabetes affecting periodontal disease status for people with sight loss from diabetic retinopathy^8^
- Challenges accessing dental care, as discussed below
- The impact of older age or disability on a patient's dentition, as older people and people with learning disabilities are more likely to develop sensory impairments. For example, the impact of polypharmacy, frailty or limited cooperation.^9^

### Hearing

Deaf people are more likely to have poor oral health, with some reports of high dental treatment needs.^10^

People who are deaf or hard of hearing have greater prevalence of hard tissue anomalies, such as enamel hypoplasia, which can be associated with common causes of hearing loss, such as prematurity and rubella.^11^ Some syndromes associated with deafness will have an impact on oral health, such as periodontal disease associated with Chediak-Higashi syndrome, Down syndrome and Papillon Lefèvre syndrome.^12^ Moreover, these syndromes can result in dental malocclusions, for example people with cleft lip and palate.^13^

A higher incidence of bruxism has been reported in people who are deaf, which is thought to fill the sensory vacuum, although this mechanism is unclear.^12^

### Visual

People with visual impairments face difficulties in maintaining good oral health.^14^ Depending on their degree of visual impairment, some patients may have difficulty identifying dental caries or gingival bleeding for example, or even more worrying, conditions such as suspicious lesions.^15^ There have been several studies reporting poor oral hygiene, gingival bleeding and dental trauma in children and adults with visual impairments.^14^^,^^16^^,^^17^ One study reported that one-fifth of visually impaired children had suffered dental trauma and almost one-third reported an oral impact on daily performances.^11^ Because they are unable to recognise early stages in oral disease, they could fail to receive prompt treatment and people with visual impairments access dental care less than people whose vision is not impaired.^16^ As well as this, they might find using some oral hygiene aids challenging, such as the use of floss.

There has been an association between some causes of ocular disease and dental caries, for example, Sjögren's syndrome.^18^ Dental anomalies secondary to congenital disease associated with sight loss may develop, for example, in syndromes such as Ehlers Danlos, Marfan or Treacher-Collins, where visual impairments and dental anomalies such as hypodontia or microdontia can occur.^19^

Similar to patients with hearing impairments, it has also been suggested that bruxism and occlusal wear may be increased in individuals with visual impairments but the exact mechanism of why this occurs is also unclear.^19^ Possibly as a consequence of this and lip or cheek biting, mucosal lesions may arise for these patients, but there is no available evidence to say this is more prevalent than the general population.^20^

### Dual impairment

Little is known about the oral health of people with dual sensory impairment. Of the studies available, most relate to the oral health of deafblind children which reported poor oral health and poor oral hygiene.^21^ One study of deafblind adults in Canada found they had poor oral health knowledge and their daily oral hygiene was minimal. Few appeared to have received dental health education and access to dental care was facilitated by the use of intervenors and a subsidised dental care programme.^15^

## Barriers to accessing dental care

Patients with disabilities and sensory impairments may face barriers when accessing dental care and these can include accessibility of the clinic and how to access dental services but will vary between patients depending on their level of impairment.^22^ Some patients with sensory disabilities may not be able to independently travel to dental clinics without additional assistance and may rely on others to aid with this. Additionally, booking appointments or receiving information about appointments may provide barriers, such as people with hearing impairments not being able to telephone to book appointments or people with visual impairments being unable to view appointment letters.^23^

People with disabilities are more likely to experience poverty and material deprivation which may impact their ability to afford treatment.^24^ Patients with sensory impairments may not be able to or may perceive that they may not be able to afford dental treatment and, depending on their circumstances, may not qualify for NHS exemptions for free dental treatment.^23^

The services that people encounter must be acceptable to those attending and if they are not, then this can provide additional barriers to care. This can be provided by ensuring that treatment is patient-centred and takes into account what each patient values. This requires an individualised approach and effective communication, which in most encounters may be achieved by reasonable adjustments.^1^

Patients with sensory impairments may require flexibility with appointment times for a number of reasons and it is important that dental surgeries are able to accommodate these adjustments. This may be due to them requiring the assistance of escorts, such as family members or support staff, or, as previously mentioned, patients with sensory impairments may have additional medical considerations that need to be taken into account and other health factors that take priority necessitating additional medical appointments.

Many patients with sensory impairments can be treated in general dental practice with reasonable adjustments; however, a small minority may require referral to more specialist services. Some dentists in general practice may lack the knowledge or skills to confidently provide care and may advise referral to specialist services, which may be an additional journey or distance from the patient's home.

Where advanced or complex sensory impairments are present, which might be alongside other comorbidities, referral to a special care dental service might be appropriate. For example, because of:

- Complex, multi-sensory impairment where communication is significantly impaired and advanced communication methods are required
- Disability where only limited cooperation is possible, where adjuncts such as sedation, general anaesthesia or clinical holding are required
- Complex multi-morbidity of ASA 3 (American Society of Anesthesiology score) or above
- A requirement for specialist equipment to access a dental chair, such as a hoist or wheelchair recliner
- Where domiciliary care is required
- Oral hygiene requires support of a third party
- Multi-disciplinary working in planning of patient care, such as with speech and language therapists
- Legal or ethical concerns, such as fluctuating or lack of capacity to consent or safeguarding concerns.

### Consent and communication

When assessing communication and capacity, it is important to work within the framework of the Mental Capacity Act (2005) and ensure that the five principles are adhered to:

1. Presumption of capacity
2. Support to make a decision
3. Ability to make unwise decisions
4. Best interest
5. Least restrictive.^25^

According to the Act, a person has capacity if they are able to understand information given to them, retain it for a sufficient period of time, weigh up information as part of the decision-making process and communicate that decision. It should not be assumed that a person with a sensory impairment does not have the capacity to make a decision and this should be assessed accordingly for each decision. The individual should be supported in making a decision themselves by all practical steps possible, including steps to aid in communication. Examples of practical steps or reasonable adjustments are described below.

## Managing patients with sensory impairments in dentistry

### Hearing impairment or deaf

When managing a patient who is deaf, there are a number of things to consider. Often patients with a hearing impairment rely heavily on their visual sense in order to aid communication. Communication will be impacted differently, depending on the degree of the patient's deafness (see Table 2).

**Table 2:** Degrees of deafness and impact on patient communication.Reproduced with permission from Dougall *et al*., 'Access to special care dentistry, part 2. Communication', *British Dental Journal*, 2008, Springer Nature^26^

Degree of deafness	Quietest sounds heard in decibels	Impact on patient
Mild	25-39	Difficulty following speech in noisy situations
Moderate	40-60	Difficulty following speech without a hearing aid
Severe	70-94	Patient may rely on lip-reading even with a hearing aid. BSL may be the preferred method of communication
Profound	≥95	BSL may be the preferred method of communication

Patients with a hearing impairment may struggle with communication, even from the waiting room.^26^ While some patients may rely on BSL and will require an interpreter to attend with them at appointments, others may be able to lipread. Others might prefer to write things down to communicate. Thus, it is vital to establish the patient's needs and preferred methods of communication at the initial consultation and ensure this is recorded in their notes, as per the AIS. It is important to face the patient directly when speaking and remove the mask, if possible, so that they can lipread. A study by Champion and Holt in 2000^27^ found that pulling the mask down away from the lips improved communication with deaf children, although with recent challenges about infection control and the increasing use of face coverings as a result of the COVID-19 pandemic, this advice may not be recommended. Clear masks are also available so that the clinician can speak to the patient, although many are only advised for consultations only, as they are not surgical grade. As with any patient, hand signals are very useful in understanding how the patient is feeling and separate signals can be used for 'stop', 'fine', etc.

Patients who are hard of hearing may use a hearing aid and high-pitched noises, such as the ultrasonic, can interfere with it. Eliminating any background noise, such as music, will facilitate communication. It may be helpful to suggest turning the hearing aid off when completing procedures that require the dental drill for the patient's comfort. If the patient is unable to hear, shouting is not advised as it can appear aggressive and lead to the patient losing confidence in the clinician. Instead, speak clearly in a loud voice and repeat the sentence, perhaps using different words to establish the same point. Lowering the pitch of your voice can also be helpful, especially as patients tend to lose high-pitch hearing first. Some people may have unilateral hearing loss and therefore will hear better if spoken to on a particular side.

Many patients will use BSL or Makaton to communicate. BSL is an official language, recognised in 2003, with its own grammar and structure and variations across regions within the UK. Makaton uses signs and symbols alongside speech and is often used by children who are hard of hearing. It may be useful to learn some basic signs in both BSL and Makaton. Not only will this aid the conversation with the patient, but it will also help to earn their trust and make them feel cared for.

There are also other aids to communicate with those with hearing loss, such as induction loops and infrared systems, as well as Typetalk systems or apps. Typetalk allows deaf people to make calls to hearing people without any need for a text phone - this is a service operated by the Royal National Institute for Deaf People.^28^ There are also other apps, such as Interpreter Now, which uses a BSL interpreter over video conferencing to interpret for telephone and face-to-face conversations. Transcribing apps can be helpful to transcribe what someone is saying to text on a phone or tablet for a deaf person to read.

Following the appointment, a leaflet can be given to the patient in order to supplement the conversations that took place within the surgery. When communication is needed with the patient outside of the practice, text messages, emails and letters are preferential to phone calls. Alternatively, with the patient's consent, a hearing next of kin or support worker could be used as a point of contact for correspondence.

Some patients with hearing loss will have been treated with cochlear implants. A cochlear implant is a small electronic device that helps provide a sense of sound to a person who is profoundly deaf or severely hard of hearing. The implant consists of an external portion that sits behind the ear and an internal portion that is surgically placed under the skin. The implant consists of:

- A microphone, which picks up sound
- A speech processor, which arranges sounds picked up by the microphone
- A transmitter and receiver/stimulator, which receives signals from the speech processor and converts them into electric impulses
- A group of electrodes that collects the impulses from the stimulator and sends them to different regions of the auditory nerve.^29^

Cochlear implants do not restore normal hearing but can give a deaf person a useful representation of sounds and ability to understand speech. These implants have some considerations within dentistry. Ideally, the speech processor should be kept at least 50 cm away and preferably out of the room during radiographic examination.^30^ Microwave diathermy, shortwave diathermy and ultrasound diathermy should not be used in these patients as they could cause damage to the implant and neurons of the inner ear.^31^ For other imaging of the head and neck, including ultrasound or magnetic resonance imaging, as well as ultrasonic scaling, patients' cochlear implant teams should be consulted before the procedure. Cone beam computed tomography, electric pulp test, panoramic radiographs and digital radiographs are safe in these patients.^30^^,^^31^ Consultation with implant teams might also be required for patients with recent implants who require inhalation sedation. Nitrous oxide diffuses into closed spaces such as the middle ear and this results in increased pressure - this can cause pain for the patient or potential damage to the inner ear.^32^

Monitoring patients with hearing impairments during conscious sedation can be challenging. Maintaining verbal feedback, as per the definition of conscious sedation, might not be possible and other communication and monitoring methods should be employed, such as the use of hand signals.^33^

### Visual impairment

Depending on the level of visual impairment, different adjustments may be required (see Table 3). It would be imperative to gain an understanding of an individual's level of visual impairment and their preferred methods of communication. When meeting them in the waiting room it may be necessary to help guide them to the dental surgery. They may have a guide dog to assist with mobility, which should be allowed into the dental surgery if needed.^34^ While a guide or assistance dog is working, they should not be touched, fed or otherwise distracted and permission should always be sought before attempting to pet the dog.^35^ When communicating with a person with a visual impairment, it is important to initially gain their attention by first speaking to them, for example by saying their name, or gently touching their arm and ensuring that you are always talking to them. Additionally, the use of verbal responses when communicating, rather than using non-verbal responses or communication, are recommended.

**Table 3:** Visual acuity and definitions of visual impairment.^26^^,^^40^Reproduced with permission from Fiske *et al*., *Special Care Dentistry: Quintessentials of Dental Practice*, Quintessence Publishing, 2009^40^

Visual acuity	Visual impairment
VA 6/12 to >6/18	Patient may not be able to drive; may have difficulty reading small print, even with glasses
VA 6/18 to >3/60	Patient is partially sighted and has a severe sight problem; can only read the top letter of the eye chart from ≤6 m
VA <3/60	Patient is legally blind; can only read the top letter of the eye chart from ≤3 m

During dental treatment, ensure the patient is told what is happening or going to happen, particularly if this will involve loud sounds or different sensations. Patients can also be given items to feel in their hands before they are used to help prepare them for a procedure, for example, an x-ray holder or impression tray.

When providing written information, such as appointment letters or reminders, oral hygiene instructions, or post-operative instructions, adaptations will be required. For people who are partially sighted, easy print information, letters and medical history forms in a large format may be all that is required. However, for those whose visual impairment is more severe, they may require additional aids, such as audio or braille; different methods are discussed in Table 4. It is important to discuss with patients what form they would find the most helpful and accessible for them and they may find that a combination of methods would be the most beneficial.^23^ It was found that visually impaired patients who were provided with oral hygiene instructions in a format of audio aids, braille and tactile models showed an improvement in plaques scores over three months and those who received a combination of all three formats showed the greatest improvement.^36^

**Table 4:** Advanced communication methods for people with sensory loss

Communication methods for sensory loss	Explanation
Clear speech and lip reading	Also known as speechreading, is a technique of understanding speech by visually interpreting the movements of the lips, face and tongue
Tadoma	Tactile lip reading where fingers are placed on the lips/jawline to feel vibrations of speech
Deafblind manual alphabet	A method of spelling out words onto a hand, with each letter denoted by a particular pattern and location on the hand
Block alphabet	Tracing block letters onto the palm of the hand
Braille	A system of raised dots that can be read with the fingers
Moon	Similar to braille but with raised curves and lines
BSL with/without adaptations: Visual frame signing Close-up signing Tactile signing Hand under hand signing Sign Supported English (SSE)	Sign language with adaptations: Making signs in a smaller area for people with smaller fields of vision In close proximity to the person Signing using touch, such as finger spelling onto a person's palm Where the receiver's hands are lightly placed upon the hands of the signer to read signs through touch and movement SSE is a form of Manually Coded English (MCE), also known as conceptually accurate signed English. Unlike BSL, this type of sign language follows the spoken and reading English language and follows its structure
Haptic communication	Non-verbal communication by touch
Makaton	Trimodal language using signs, symbols and speech
Symbol systems	Using symbols or pictures to communicate
Objects of reference	An object that can be held or touched and is used to represent an activity, a person, a place or even a concept, for example, a person may hold up their favourite cup to indicate they would like a drink
Pictorial communication systems eg Widgit	A symbol-based language using pictorial symbols, either as an alternative to text, or to accompany it
Note taking	Writing words down
Electronic communication (with braille output or large font on screen)	Using technology such as tablets to display large text, or with braille or audio outputs
Technology to aid communication	Apps such as transcribing apps, or using symbols alongside words or text, such as MyChoicePad
Individuals' own personal signs	Signs individual to the person that do not follow an official sign language
Large print (font size 16 or above)	Text in larger typeface
Low vision aids (magnifiers, task lighting)	Illumination or magnification aids
Other technology systems	Speech recognition systems, speech synthesisers

Adaptations to the surgery and environment may be useful, for example, the use of bright colours for edges of steps, doors and pillars.^37^ Being mindful of background noise, such as the radio, is also important, as patients with visual impairments will rely more heavily on other senses, such as hearing or tactile sensations. Due to an increase in tactile sensitivity, patients with visual impairments may have an intolerance to or difficulty adjusting to removable prostheses and a training plate may aid with desensitisation when these are being considered.^38^ Additionally, some patients with visual impairments may also experience a lower tolerance to pain and therefore effective analgesia during dental treatment is essential.^39^

### Dual impairment

The impact of dual sensory impairment on an individual will vary according to the learning opportunities they have had.^40^ People who are born deafblind may have little or no formal language and only limited understanding of the world because they have never been able to watch or listen to other people. However, people who acquire dual sensory impairment may remember sight and/or hearing and therefore are more likely to have some language learning.

Communication with people who are deafblind can be challenging, particularly for those with congenital impairments. Advanced methods of receptive and expressive communication might be required in liaison with a family member, carer or speech and language therapist, often in combination. Some of these methods can be seen in Table 4.

## Conclusion

Sensory impairments are common within the UK and dental practitioners have a legislative duty to make reasonable adjustments to dental care for this population, including considering alternative communication methods. The oral health of this group of patients is often poorer than the general population and they face significant barriers to accessing dental care. Simple adjustments can be implemented in general dental practice to manage these patients; however, when more advanced adjustments are required or associated comorbidities affect dental care, referral to special care dentistry might be appropriate.^41^
